# Functionalization of the *TMEM175* p.M393T variant as a risk factor for Parkinson disease

**DOI:** 10.1093/hmg/ddz136

**Published:** 2019-06-07

**Authors:** Sarah Jinn, Cornelis Blauwendraat, Dawn Toolan, Cheryl A Gretzula, Robert E Drolet, Sean Smith, Mike A Nalls, Jacob Marcus, Andrew B Singleton, David J Stone

**Affiliations:** 1 Merck & Co., Inc., West Point, PA 19486, USA; 2 Laboratory of Neurogenetics, National Institute on Aging, National Institutes of Health, Bethesda, MD, USA; 3 Data Tecnica International, Glen Echo, MD, USA

## Abstract

Multiple genome-wide association studies (GWAS) in Parkinson disease (PD) have identified a signal at chromosome 4p16.3; however, the causal variant has not been established for this locus. Deep investigation of the region resulted in one identified variant, the rs34311866 missense SNP (p.M393T) in *TMEM175*, which is 20 orders of magnitude more significant than any other SNP in the region. Because *TMEM175* is a lysosomal gene that has been shown to influence α-synuclein phosphorylation and autophagy, the p.M393T variant is an attractive candidate, and we have examined its effect on TMEM175 protein and PD-related biology. After knocking down each of the genes located under the GWAS peak via multiple shRNAs, only *TMEM175* was found to consistently influence accumulation of phosphorylated α-synuclein (p-α-syn). Examination of the p.M393T variant showed effects on TMEM175 function that were intermediate between the wild-type (WT) and knockout phenotypes, with reduced regulation of lysosomal pH in response to starvation and minor changes in clearance of autophagy substrates, reduced lysosomal localization, and increased accumulation of p-α-syn. Finally, overexpression of WT TMEM175 protein reduced p-α-syn, while overexpression of the p.M393T variant resulted in no change in α-synuclein phosphorylation. These results suggest that the main signal in the chromosome 4p16.3 PD risk locus is driven by the *TMEM175* p.M393T variant. Modulation of TMEM175 may impact α-synuclein biology and therefore may be a rational therapeutic strategy for PD.

## Introduction

Parkinson disease (PD) is the most common neurodegenerative movement disorder, affecting over 6 million people worldwide and resulting in over 211 000 deaths per year globally (1). While therapeutics that temporarily alleviate the symptoms of PD (e.g. l-Dopa) exist, there are currently no treatments that can slow or stop the course of disease progression. An understanding of the environmental and genetic factors contributing to disease pathophysiology will be critical for the development of disease-modification therapies. Toward this end, several large genetic studies have been completed to identify genetic risk factors for PD. From these studies, cellular pathways associated with genes that confer PD risk are beginning to emerge (2); however, for genome-wide association studies (GWAS), the majority of effector variants or even effector gene have not yet been identified. Since many genes can reside near the location of GWAS signals, the identification of the genetic variants, which drive GWAS signals, is critical for both an understanding of disease physiology and drug development.

Recent large-scale meta-analyses of PD GWAS have described a highly significant peak at chromosome 4 (latest PD GWAS, chr4:951947 (hg19)/rs34311866, *P* = 1.47*E* − 50, odds ratio [OR] = 1.232, standard error [SE] = 0.014) (3, 4). Multiple genes located under this peak are considered candidates due to their functional roles in cellular processes implicated in the pathophysiology of PD, including cyclin G-associated kinase (*GAK*), complexin 1 (*CPLX1*), and transmembrane protein 175 (*TMEM175*). GAK is a known binding partner of LRRK2, a well-established genetic factor in both sporadic and familial PD (5). *Cplx1* knockout (KO) in mice results in both an ataxia phenotype (6) and damage to the nigrostriatal pathway (7). TMEM175 has recently been identified as a novel lysosomal potassium channel that is involved in regulation of lysosomal pH and autophagy (8), as well as α-synuclein aggregation and mitochondrial function (9). Importantly, conditional analysis on the lead SNP (rs34311866) has demonstrated an independent second signal at this locus, raising the possibility that there may be more than one gene responsible for the association with PD in this genomic region (3, 4).

The lead SNP (rs34311866) is a missense variant, resulting in a methionine to threonine conversion in the 10th transmembrane domain of *TMEM175* in exon 11 (p.M393T). TMEM175 is a protein of high interest for several reasons. First, TMEM175 is localized to the lysosomal membrane, and known genetic risk factors and causal genes for PD include variants in other genes involved in lysosomal pathway such as glucocerebrosidase (*GBA*) (10), *ATP13A2* (11), and *GALC* (4). Second, the crystal structure of TMEM175 has been published (12) and suggests that it may be a gated ion channel, and therefore potentially modulated by pharmacological agents. Finally, the effect of *TMEM175* KO on synuclein aggregation demonstrates a link to the primary neuropathology of PD, increasing the probability that modulation of channel function may have efficacy in sporadic PD. While this is an attractive and testable candidate for mechanism of pathogenicity, to date, the p.M393T variant has not been examined and it is unclear if this mutation affects protein function in any meaningful way.

We have performed an in-depth analysis of the PD chromosome 4p16.3 locus, which suggests that the rs34311866/p.M393T SNP is the most significant variant in the region and quite possibly the variant driving PD risk. Knockdown (KD) via shRNA of all genes in this locus demonstrated that *TMEM175* was the only gene consistently associated with changes in levels of phosphorylated α-synuclein (p-α-syn). Functional studies on the p.M393T variant showed changes similar to KO, although less severe in magnitude, and reduced lysosomal expression of TMEM175 protein. Finally, overexpression of wild type TMEM175 resulted in a reduction of p-α-syn *in vitro*. Overexpression of the p.M393T variant failed to reduce p-α-syn levels, even when lysosomal levels were increased above normal wild-type (WT) levels. Taken together, these data suggest that the *TMEM175* p.M393T variant is responsible for the main signal in the chromosome 4p16.3 locus and that this variant therefore confers risk for PD. TMEM175 modulation may be a rational therapeutic strategy to reduce synuclein pathology in PD.

## Results

### TMEM175 Locus overview

**Figure 1:**
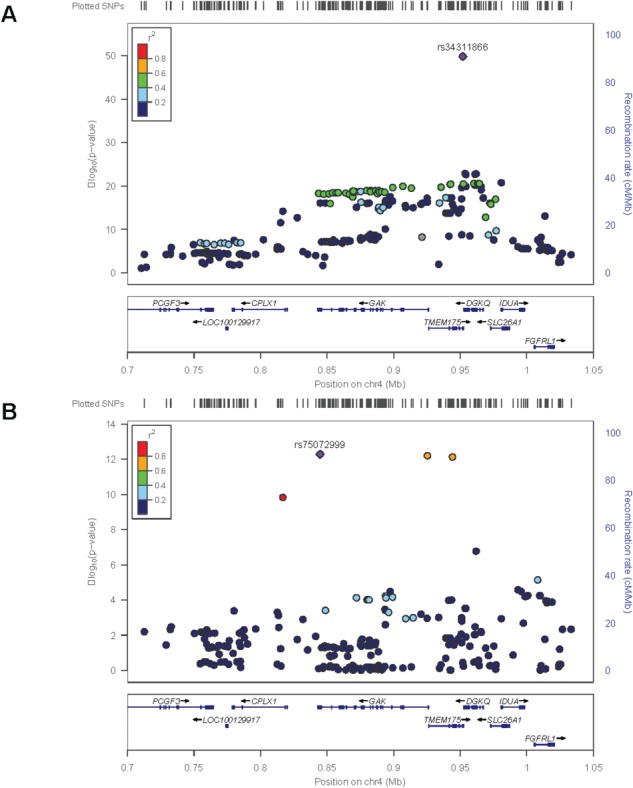
PD GWAS *TMEM175* locus overview. (**A**) *TMEM175* locus association signal is plotted, rs34311866 is the main signal, and no other variants in high LD are observed. (**B**) Conditional analysis on the main signal (rs34311866) identified a secondary genome-wide significant haplotype of 4 variants.

Examining the TMEM175 locus PD GWAS signal found the rs34311866 missense SNP (p.M393T) in TMEM175 to be the most significant variant in the region by over 20 orders of magnitude (*P* = 1.47*E* − 50, OR = 1.232, SE = 0.014) (Fig. 1A). Interestingly, no other variants in high linkage disequilibrium (LD) (*R*^2^ < 0.6) are observed, further suggesting p.M393T as the obvious candidate for causal variant. Both the additive model and recessive model were investigated to identify potential, and as expected, there was no significant difference between the recessive model and the additive model with two rs34311866 alternative alleles (OR = 1.528 and OR = 1.518, respectively). Conditional analysis on the main signal (rs34311866) identified a secondary genome-wide significant signal with a protective effect (Fig. 1B), consisting of at least four variants in high LD (*R*^2^ > 0.6), as previously shown (3, 4). Annotation of these four variants showed that there was one intronic variant in CPLX1, one synonymous variant in GAK (p.P66P), one intronic variant in TMEM175 and one coding variant in TMEM175 (p.Q65P) (Table 1). No other coding variants in TMEM175, GAK, or any other gene in the locus were significantly associated with PD. Additionally, no brain eQTLs were identified in the GTEX database (https://gtexportal.org/) neither for any of these four variants nor for the TMEM175 p.M393T variant.

**Table 1:** PD GWAS chromosome 4p16.3 locus variant overview with both initial hit and conditional hit variants

Rs-ID	Chr	bp	RefA	Freq	*b*	SE	*P*-meta	*P*-conditional	Variant type	Note
rs34311866	4	951 947	C	0.1941	0.208639	0.0153	1.47*E* − 50	na	Coding in *TMEM175* p.M393T	Normal top hit
rs34884217	4	944 210	C	0.0931	−0.215672	0.0262	1.55*E* − 16	7.42*E* − 13	Coding in *TMEM175* Q65P	Conditional hit^b^
rs873786	4	925 376	T	0.0968	−0.211956	0.0256	5.06*E* − 17	6.30*E* − 13	Intronic in *TMEM175* and *GAK*	Conditional hit
rs75072999	4	844 781	A	0.0863	−0.219401	0.0268	8.43*E* − 17	5.17*E* − 13	Coding in *GAK* but synonymous^a^	Conditional hit
rs76444973	4	816 756	T	0.0703	−0.243346	0.0318	6.57*E* − 15	1.48*E* − 10	Intronic in *CPLX1*	Conditional hit

^a^This variant can be reported to be missense, but it appears only to be a missense SNP in a very short transcript ENST00000504668, which is annotated as “Nonsense mediated decay” in Ensembl.

^b^Chang *et al.* (4) nominated conditional hit.

Chr, chromosome; bp, base pair based on hg19 build; ref A, reference allele; freq, allele frequency; *b*, beta; SE, standard error; *P*-meta, *P*-value of the meta-analysis; *P*-conditional, *P*-value of the conditional analysis on rs34311866; na, not applicable.

### TMEM175 deficiency alone exacerbates PFF-induced p-α-syn in rat primary hippocampal neurons

**Figure 2:**
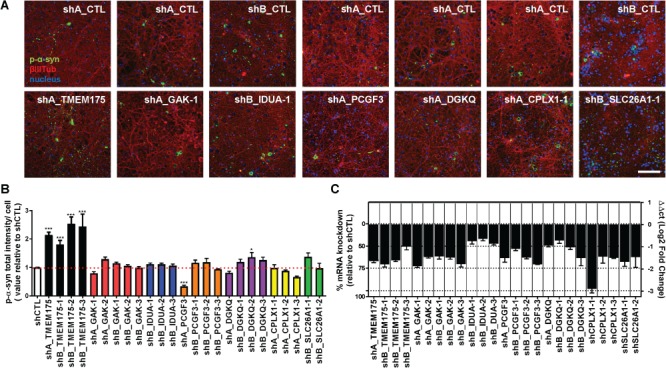
*TMEM175* deficiency alone exacerbates PFF-induced p-α-syn in rat primary hippocampal neurons. (**A**) Representative images from immunocytochemistry staining of p-α-syn inclusions (green) and βIII-tubulin (red) at 14-day PFF treatment in control (shCTL) and target shRNAs (shTM175, shGAK, shIDUA, shPCGF3, shDGKQ, shCPLX1, and shSLC26A1; shA, Sigma, MISSION Lentiviral Transduction Particle; shB, Dharmacon, SMARTvector Lentiviral shRNA) infected neurons. Blue indicates nuclear staining by Hoechst. One representative pair of images (shCTL and shTarget) out of multiple shRNAs targeting non-overlapping regions within each gene is shown. Scale bar, 100 μm. (**B**) Intensity of p-α-syn inclusion relative to control (shCTL) was plotted for each shRNAs (number denotes one shRNA out of multiple targeting different regions within the same target) (n = 6). (**C**) % KD (left) and ΔΔct values of each gene's mRNA (right) after transduction with shRNAs relative to control shRNA (shCTL) measured by qRT-PCR. Data presented as mean + standard error of the mean. One-way ANOVA in (B). ^*^P < 0.05 and ^***^P < 0.001. shCTL vs. shRNAs against each indicated gene.

Previously, we have shown that reduction of TMEM175 by KO or KD leads to increased accumulation of p-α-syn inclusions in the preformed fibril (PFF) α-synuclein model (9). In this model, endogenous α-synuclein is required for PFF to seed and sustain the formation of hyperphosphorylated α-synuclein aggregates (13, 14) in time- and dose-dependent manner (Fig. S1A–C). To determine if other genes under the chromosome 4 GWAS peak could play a role in α-synuclein biology, we utilized the same PFF α-synuclein model (13, 15) in rat hippocampal neuronal cultures as previously described (9). Rat hippocampal neurons were seeded with human α-synuclein PFF 4-day preinfection with lentiviral control shRNA (shCTL) or shRNAs against *TMEM175*, *GAK*, *IDUA*, *PCGF3*, *DGKQ*, *CPLX1*, and *SLC26A1* and incubated for additional 14 days, after which they were evaluated for the intensity of p-α-syn by immunocytochemistry. Multiple distinct shRNAs targeting different regions of each gene were tested, and shRNAs that achieved at least 50% KD without affecting cell viability were chosen for analysis. The amount internalized PFF after 4 days of shRNA against each gene was not changed much compared with control shRNA, and converged to a same extent at 24 h, when all treatment groups show similar amounts of total internalized PFF (Fig. S1D). As previously shown, *TMEM175*-KD resulted in a significant 2- to 3-fold increase in p-α-syn staining compared to control (Fig. 2A and B) and was consistent across distinct shRNAs. However, shRNAs against other genes with KD of the target mRNA to levels comparable with that of *TMEM175* shRNAs did not lead to significant change in p-α-syn (Fig. 2A, B and C). In most cases, there was no change at all, and in other cases, the direction of change was not consistent across multiple shRNAs. For example, one shRNA against *GAK* that lead to ~60% KD of mRNA (Fig. 2B and C, shA_GAK1) resulted in ~1.3-fold increase in p-α-syn intensity, while other shRNAs that caused a ~60% or higher KD (Fig. 2B and C, shA_GAK-1, shB_GAK-1, -2 and -3) result in no changes p-α-syn. In other cases, knocking down target mRNA ~ 0% or more did not result in changes in the level of p-α-syn (Fig. 2B and C, shPCGF3s and shCPLXs). While mRNAs of *DGKQ* and *IDUA* could not be reduced more than 50% because of cytotoxicity, this 50% KD did not lead to changes in the level of p-α-syn (Fig. 2B and C, shDGKQs and shIDUAs), whereas 50% KD in *TMEM175* still resulted in significant accumulation of p-α-syn (Fig. 2A and B, shB_TM175-3). Together, these results suggest that only *TMEM175* deficiency consistently influences p-α-syn levels in the PFF-induced α-synuclein pathology model.

### TMEM175 overexpression reduces PFF-induced p-α-syn inclusions

**Figure 3:**
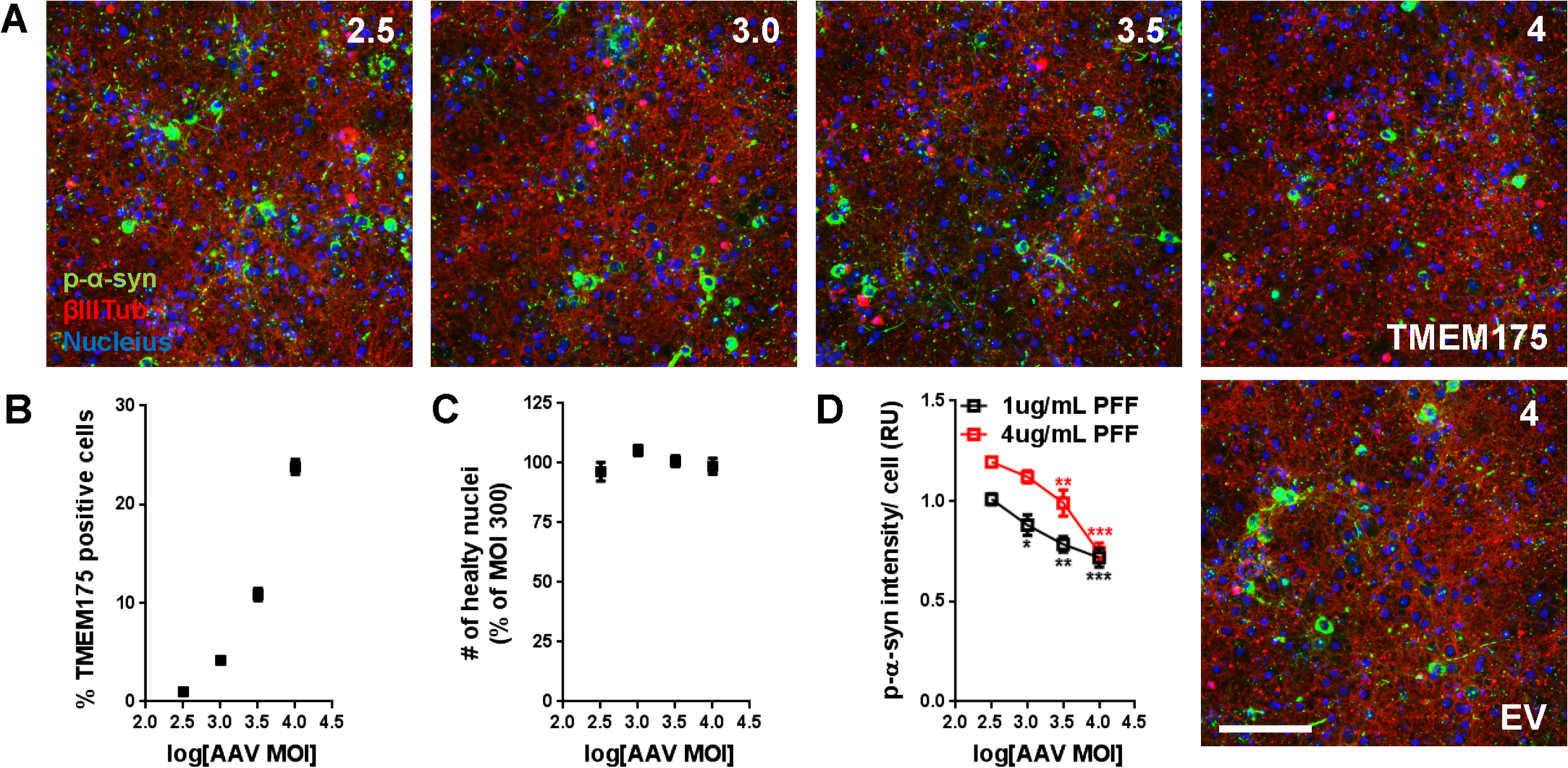
TMEM175 overexpression reduces PFF-induced p-α-syn inclusions. (**A**) Immunocytochemistry staining of p-α-syn inclusions (green) and βIII-tubulin (red) at 14-day PFF treatment in neurons treated with increasing doses of AAV TMEM175. Blue indicates nuclear staining by Hoechst. Scale bar, 100 μm. (**B–D**) Percentage of TMEM175 positive cells (B), number of healthy nuclei (C), and relative intensity of p-α-syn inclusion in TMEM175 positive cells against increasing MOI (D) was plotted (n = 8). Data presented as mean + standard error of the mean. One-way ANOVA in (D). ^*^P < 0.05, ^**^P < 0.01 and ^***^P < 0.001. log [MOI] 2.5 vs. log [MOI] 3, 3.5 and 4.

Since decreasing TMEM175 expression lead to elevation of p-α-syn, we asked whether increasing TMEM175 expression could have an opposite effect. We generated adeno-associated virus 8 (AAV8) expressing empty vector (EV) and WT *TMEM175*. Following preinfection with increasing multiplicity of infection (MOI) of AAVs, rat primary hippocampal neurons were incubated with PFF for 14 days, after which they were evaluated for the intensity of p-α-syn inclusions by immunocytochemistry. Increasing MOI from 300 to 10 000 led to increasing number of TMEM175-positive cells (Fig. 3B), and increasing expression of TMEM175 (Fig. 5A, WT TMEM175). A minimum dose of 300 MOI WT *TMEM175* AAV resulted in similar intensity of p-α-syn as empty AAV control (Fig. 3A, 2.5 vs. EV). As the number of cells positive for TMEM175 expression increased, the total intensity of p-α-syn inclusions decreased in a MOI-dependent manner. This result suggests that there is directionality from TMEM175 loss of function to TMEM175 gain-of-function, which modulates the amount of p-α-syn inclusions.

### TMEM175 p.M393T mutation partially phenocopies *TMEM175* KO

**Figure 4:**
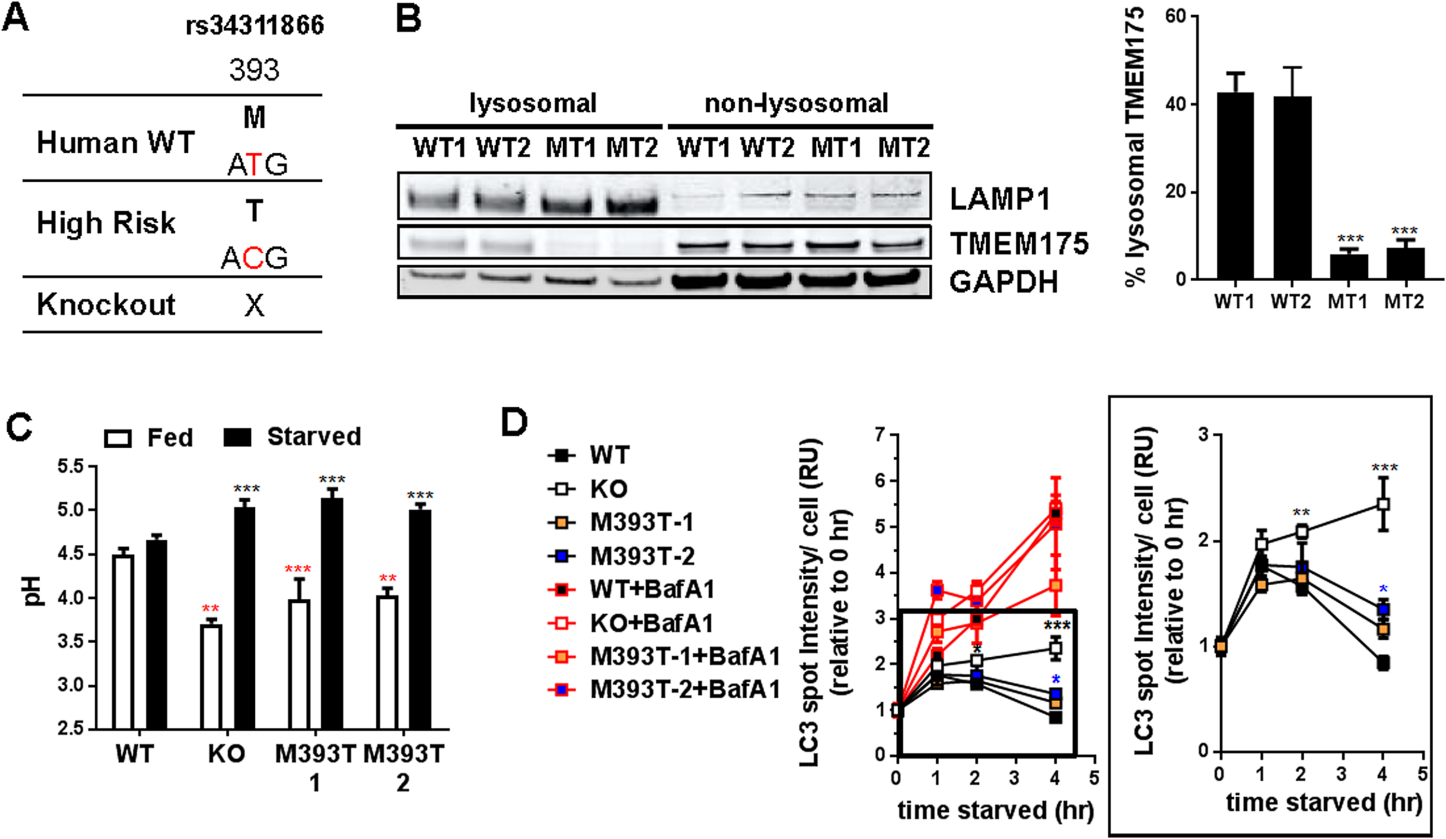
TMEM175 p.M393T mutation produces similar lysosomal pH and autophagosome clearance phenotypes as TMEM175 KO. (**A**) Description of CRISPR engineered SH-SY5Y p.M393T TMEM175 cells. (**B**) Amount of TMEM175 in lysosomal and non-lysosomal fraction of wild type (WT) and p.M393T(MT) cells was assessed by western blotting. Quantified amounts relative to WT is shown on the right (n = 4). (**C**) Lysosomal pH of WT, TMEM175 KO, and two independent clones carrying p.M393T TMEM175 were measured in fed (white) and starved (stv, black, EBSS 4 h) conditions (n = 9). (**D**) Autophagosome-associated LC3 puncta from SH-SY5Y WT, TMEM175 KO, and p.M393T TMEM175 clones were assessed by immunohistochemistry at indicated time points after starvation. Quantified puncta intensities are shown (n = 3). Data presented are mean + standard error of the mean. One-way ANOVA (B). Two-way ANOVA (C–D). ^**^P < 0.01 and ^***^P < 0.001 WT vs. KO; WT vs. p.M393T-1 and WT vs. p.M393T-2.

Previously, we have demonstrated that *TMEM175* KO destabilized lysosomal pH in the SH-SY5Y neuroblastoma cell line. To see if the p.M393T mutation had a functional effect on TMEM175 modulation of lysosomal pH, the p.M393T SNP was engineered into the SH-SY5Y neuroblastoma cell line using the CRISPR-Cas9 nickase approach. Two clonal cell lines p.M393T-1 and p.M393T-2 were generated (Fig. 4A). The level of lysosomal TMEM175 was significantly decreased in the two clones harboring p.M393T mutation relative to WT (Fig. 4B). Under the fed condition, lysosomal pH of *TMEM175* KO and p.M393T SH-SY5Ys was slightly more acidic than WT (Fig. 4C, white bar). Upon serum starvation, a significant increase in lysosomal pH was observed in both *TMEM175* KO and p.M393T cells (p.M393T-1 and 2), while WT SH-SY5Y cells maintained lysosomal pH in both fed and starved conditions (Fig. 4C, black bar). We also observed ~10% loss by rate constant of GBA activity in lysosomal fraction from M393T clones relative to WT (Supplementary Material, Fig. S2), which is milder than ~30% loss observed in TMEM175 KO (9). To further investigate whether p.M393T mutation also interfered with the completion of autophagy similar to *TMEM175* KO, the clearance of autophagy substrates was assessed by following the levels of microtubule-associated *protein* 1A/1B-*light chain 3-II* (LC3-II), at multiple time points after starvation with or without bafilomycin A treatment using immunocytochemistry. In the absence of bafilomycin A, one CRISPR-generated p.M393T line showed delayed degradation of LC3 as in *TMEM175* KO cells, whereas the other CRISPR-generated p.M393T line showed a small, non-significant trend in accumulation of LC3 puncta relative to WT 4 h after starvation (Fig. 4D), indicating that the effect of the p.M393T mutation on autophagy control is less severe than that of *TMEM175* KO. Adding bafilomycin A resulted in accumulation of LC3 over time after the initiation of starvation to the same degree in all cells (Fig. 4D, red line). Along with incomplete lysosomal mislocalization and mild decrease GBA activity, these results suggest TMEM175 p.M393T as partial loss-of-function mutation.

### TMEM175 p.M393T overexpression does not reduce p-α-syn inclusions

**Figure 5:**
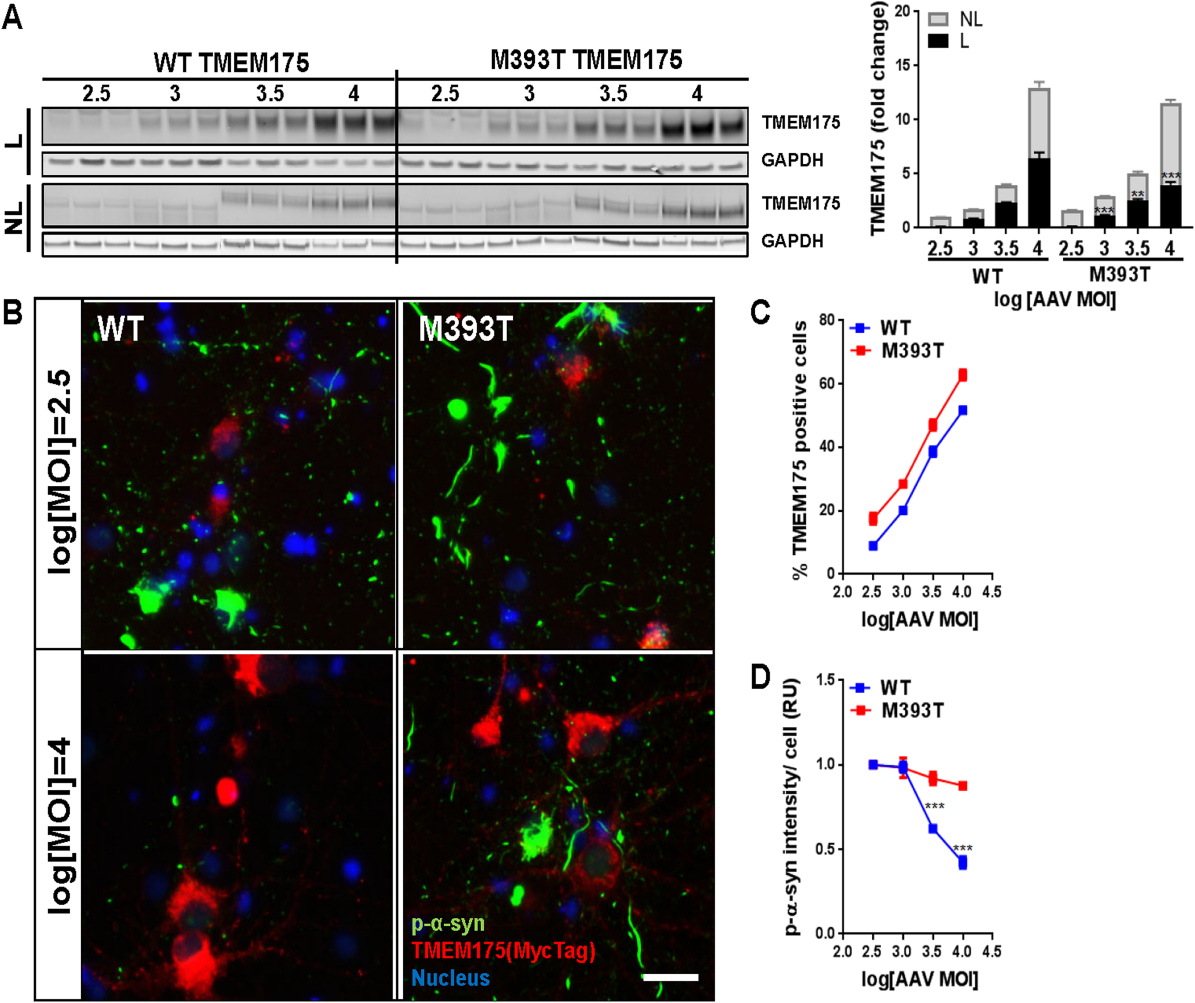
TMEM175 p.M393T overexpression has no effect on PFF-induced p-α-syn. (**A**) Amount of ectopically expressed WT or p.M393T TMEM175 in lysosomal (L, black in graph) and non-lysosomal (NL, grey in graph) fraction was assessed by western blotting. Quantified amounts are shown as fold change relative to WT MOI 300 on the right (n = 3). (**B**) Immunocytochemistry staining of p-α-syn inclusions (green) and ectopically expressed TMEM175s (red, MycTag) at 14-day PFF treatment in WT TMEM175 (WT) and p.M393T TMEM175 (p.M393T) infected neurons. Blue indicates nuclear staining by Hoechst. Scale bar, 25 μm. (**C**, **D**) Percentage of TMEM175 positive cells (C) and intensity of p-α-syn inclusion in overexpression-positive cells (D) against increasing MOI were plotted relative to MOI of 300 (n = 4). Data presented are mean + standard error of the mean. One-way ANOVA, WT vs. p.M393T for each MOI (A). One-way ANOVA, Log [MOI] 2.5 vs. log [MOI] 3, 3.5 and 4 in each genotype (D). ^**^P < 0.01 and ^***^P < 0.001.

To determine if apparent partial loss-of-function caused by p.M393T mutation also influence TMEM175 ability to ameliorate α-synuclein phosphorylation, primary hippocampal neurons were treated with increasing MOI of p.M393T TMEM175 AAVs, incubated with PFF for additional 14 days, and evaluated for protein expression and the amount of p-α-syn inclusions. In parallel, control neurons were treated with WT TMEM175 AAVs. While increasing dose of AAV led to corresponding higher levels of total TMEM175 for both WT and p.M393T, the amount of lysosomal TMEM175 was significantly reduced for p.M393T than WT at each dose (Fig. 5A). This was in line with p.M393T clones having less lysosomal TMEM175, suggesting that the mutation alters the localization of the protein. In a similar way, while increasing dose of WT TMEM175 significantly reduced the intensity of p-α-syn inclusions, p.M393T TMEM175 did not result in a significant decrease in the amount of p-α-syn inclusions (Fig. 5B and D). This was true even at the highest dose (log [MOI] = 4) of AAV where both WT and p.M393T TMEM175 lead to ~60% TMEM175 positive cells, but only WT TMEM175 induced ~50% decrease in p-α-syn relative to lowest dose (log [MOI] = 2.5) (Fig. 5C and D). Together, these results suggest that the reduced-function-like phenotypes in the TMEM175 p.M393T variant originate from both inefficient lysosomal localization and altered protein function.

## Discussion

The chromosome 4p16.3 GWAS locus is the only one of the 4 most significant PD genetic signals that has remained “unsolved” in that there is no clear candidate causal gene. The top four GWAS signals include two well-established protein components of neuropathological features of PD: *SNCA* (encoding α-synuclein), the major component of Lewy bodies (16) and *MAPT* (encoding tau), which is present in the form of neurofibrillary tangles in the substantia nigra of roughly 80% of PD patients (17). Furthermore, the fourth most significant signal is believed to be driven by *GBA* (glucocerebrocidase), an established risk factor for PD and Gaucher disease (10). Here we provide evidence suggesting that the chromosome 4p16.3 GWAS signal is driven by the missense SNP (p.M393T) in *TMEM175*, which is the most significant variant in the region by over 20 orders of magnitude. A survey of genes in the region showed that only *TMEM175* increased α-synuclein phosphorylation upon KD in the α-synuclein PFF model, and cells edited via CRISPR to be homozygous for the p.M393T variant showed impaired regulation of lysosomal pH, reduced lysosomal localization of TMEM175, and increased α-synuclein phosphorylation. Because changes in autophagy were only significant in one line, the p.M393T mutation does not appear to affect autophagy to the extent of *TMEM175* KO. Further work in other cell lines and primary neurons will be necessary to determine if the small effect seen in p.M393T-2 line represents a true, small effect on autophagy control with this mutation. Importantly, conditional analysis of the GWAS signal (controlled for the p.M393T variant) clearly shows a secondary signal, which is a haplotype of 4 variants. This region on chromosome 4p16.3 is further complicated by the fact that GAK and TMEM175 share the same promoter region and therefore are likely to be co-regulated. While the p.P65Q variant in TMEM175 is an attractive candidate for the driver of the secondary signal, at this time, it is not possible to rule out other genes with strong mechanistic connections to PD such as *GAK* and *CPLX1*.

Although we used a large imputation reference panel derived from over 38 000 individuals, it is likely that some of genetic variation was missed. Indels, repeat expansions and large structural variants are not included in imputation reference panels (Haplotype Reference Consortium); therefore, we cannot comment on the influence of those on the GWAS signal in this region. Future larger reference panels based on high coverage whole genome sequencing probably will improve this.

The p.M393T variant was demonstrated here to result in reduced lysosomal localization of protein, although the absolute cellular level of TMEM175 protein was unaffected. While it is tempting to hypothesize that this reduced lysosomal expression is driving the increased risk of PD with the variant, it is important to note that in the highest level of overexpression achieved for the p.M393T variant (log [MOI] 4, which did not reduce p-α-syn), the lysosomal level of TMEM175 protein was higher than the level of lysosomal TMEM175 protein seen for the intermediate WT overexpression (log [MOI] 3.5), which significantly reduced p-α-syn. Therefore, differences in lysosomal α-synuclein processing between the WT and p.M393T variant do not appear to be driven solely by lysosomal protein levels.

There are several possible cellular pathophysiological mechanisms through which the p.M393T variant may increase PD risk, including impact to lysosomal function, regulation of autophagy, or synuclein aggregation. These mechanisms are, of course, not mutually exclusive, and may be components of a larger pathway driving disease risk. Synuclein aggregation is of special interest due to its genetic connection with the late-onset, sporadic form of PD. Alzheimer disease (AD) is characterized by senile plaques comprised primarily of β-amyloid (Aβ) and hyperphosphorylated tau in the form of neurofibrillary tangles; however, the largest AD GWAS have not shown a genetic signal in the regions of *APP* or *MAPT* in late-onset AD (18). Similarly, while cytoplasmic TDP-43 inclusions are a hallmark of amyotrophic lateral sclerosis (ALS), and rare pathogenic variants in the *TARDBP* gene have been linked to ALS (19–21), to date, no large GWAS or meta-analysis of ALS GWAS has detected a signal near the *TARDBP* gene (22, 23). A notable difference for PD is that for late-onset, sporadic disease the top GWAS signal is associated with *SNCA*; the main protein component of Lewy bodies, which are the primary cellular neuropathology of the disease. Therefore, while therapies targeting Aβ in late-onset AD have largely failed, targeting of α-synuclein in PD may be more likely to slow disease progression due to the direct genetic connection between α-synuclein and disease risk. Indeed, a recent GWAS on age at onset for PD found only 2 genome-wide significant peaks: *SNCA* and the *TMEM175* p.M393T variant (24).

Analysis of the crystal structure of the TMEM175 potassium channel suggests that it has both an open and closed state (12, 25), raising the possibility that it may be a gated ion channel. Before attempting the development of compounds for pharmacologic modulation of TMEM175, it is critical to assess the complete directionality of effect. That is, while reduction of protein expression (through KO or KD) may increase α-synuclein phosphorylation and disease risk, upregulation of protein levels or activity may not conversely reduce synuclein pathology and disease risk. Indeed, it is possible that there may be an “optimal zone” for TMEM175 activity, and movement out of this range by either increased or decreased activity may be deleterious. The data presented here, both genetic and functional, suggest that this is not case, and modulation of activity may be an effective route to disease-modification therapy. First, overexpression of WT TMEM175 protein levels significantly reduced α-synuclein phosphorylation. While this effect was not observed with the p.M393T variant, most PD patients will have at least one copy of the WT allele, and increased activity could be expected to slow α-synuclein aggregation. Second, one of the candidates for the conditional GWAS signal is the p.Q65P variant, which appears to have a protective effect. This again suggests that TMEM175 is not optimized in most individuals for maximal suppression of α-synuclein pathology. A critical caveat is that in this study all measurements of α-synuclein phosphorylation were made in an in *vitro* model, and it is not known how faithfully this models the true state of α-synuclein biology in the diseased human brain.

Significant additional study is required to determine if TMEM175 is suitable as a target for PD disease-modification therapy. First, it will be critical to determine if changes in channel activity, and not some other pleiotropic activity of the protein, are driving differences between the WT and p.M393T variants on lysosomal function, autophagy control, and α-synuclein phosphorylation. Second, TMEM175 differs from canonical K^+^ channels, and the specific channel properties are not fully understood. The cellular signaling pathways that influence channel activity need to be identified and examined. Finally, the causal variants behind the conditional GWAS signal at this site need to be identified to fully understand if other TMEM175 variants at this locus influence PD risk or if other genes are involved.

## Materials and Methods

### Genetic analyses

PD GWAS summary statistics were obtained from previously published GWAS (3, 4). Conditional analyses were performed on the summary statistics using GCTA COJO (26, 27) with rs34311866 as conditional SNPS and using a large reference panel of 20 K cases and 20K controls derived from International Parkinson Disease Genomics Consortium (IPDGC) cohort as described previously (24, 28). Additional genetic analyses were performed on the IPDGC cohorts as described previously (28) using PLINK 1.9 (29). All high-quality (*R*^2^ > 0.8) imputed missense variants in TMEM175 and GAK were tested for association with PD using logistic regression and PC1-20 as covariates. No missense variants in CPLX1 were imputed. For the lead SNP rs34311866, both an additive and recessive model were tested. Locus association plots were created using LocusZoom (30).

### Cell culture

SH-SY5Y (31) cells were maintained in DMEM+F12 (Gibco) supplemented with 10% HI-FBS (Invitrogen), 0.5 mg/ml zeocin (Invitrogen), 7.5 μg/ml Blasticidin (Invitrogen) and 1× antibiotics (100 units/ml penicillin and 100 μg/ml streptomycin, Invitrogen). For culturing primary rat hippocampal neurons, dissected hippocampus from embryonic day 18 rats (Brain Bits, hp) was used. For the study of αSyn KO, dissected cortices from embryonic day 18 WT and αSyn KO C57BL/6 mouse were used. Tissues were transferred to 50 ml conical tube and left to settle. Twenty milliliters of prewarmed Dulbecco's Phosphate-Buffered Saline (DPBS, Gibco) was added to the tissue to wash for 1 min with gentle mixing. After DPBS removal, the tissue was incubated in 3 ml of TrypLE Select (Gibco) at 37°C water bath for 20 min with occasional flicking to stir up the settled material. After incubation, 1 ml of media was added, and tissue was manually triturated with pipetting. Dissociated cells were spun down at 650*g*, and the pellet was resuspended in 10 ml of Neurobasal A (Gibco) medium supplemented with 1× B27 (Gibco), 1× GlutaMax (Gibco) and antibiotics (100 units/ml penicillin and 100 μg/ml streptomycin, Gibco), passed through a 100 μm cell strainer and plated onto poly-d-lysine-coated 96-well plate (Biocoat). Cells were fed weekly by 50% media change.

### Generation of p.M393T TMEM175 with CRISPR

To introduce p.M393T (ATG > ACG) SNP modification in the TMEM175 gene using CRIPSR nickase technology in SH-SY5Y cells, guide RNA pair targeting 5′ GCCACGTGGACCACGGCGCTGC 3′ (L) and 5′ CCGAGGGCTGCAGCGTCTCCGC3′ (R) was transfected with WT Cas9 nicking nuclease via nucleofection. Individual clones were diluted from transfected population, isolated, expanded, and screened for (ATG > ACG) TMEM175 modification by next generation sequencing, restriction fragment length polymorphism, and Sanger sequencing.

### Lysosome pH imaging and lysosomal staining

Lysosome pH measurement was adapted from a previously described method (8, 9). Cells plated on 96 well plates were loaded overnight with 250 μg/ml Oregon-Green 488-conjugated Dextran (Invitrogen) and chased with media (or EBSS for starvation) for 3 h before imaging. Cells were washed with live cell imaging solution (Invitrogen) and imaged by ArrayScan High Content Platform (Thermofisher). The solution used for imaging starved cells contained (in mm) 140 NaCl, 2.5 KCl, 1.8 CaCl2, 1 MgCl2, and 20 HEPES (pH 7.4), and for the non-starvation groups, 1× amino acids (made from 50× amino acids mixture, Life Technologies) was added. Fluorescence was imaged using ArrayScan™ XTI Live High Content Platform (Thermofisher) with a 20× objective. In situ pH calibrations were performed at the end of imaging for each well using Intracellular pH Calibration Buffer Kit (Invitrogen) in which Isotonic K+ solutions supplemented with 10 μM of nigericin and valinomycin with pH ranging from 4.5 to 6.5 were used as pH standard solutions. The resulting fluorescence intensity was used to obtain the pH standard curve for each group. Fluorescence intensity was quantified from Oregon-Green 488-positive puncta from images using HCS Studio™ 2.0 Cell Analysis Software's compartmental analysis BioApplication. The pH value for each group was calculated by fitting the measured average fluorescence intensity of each group to pH standard curve generated from *in situ* pH calibrations from each group.

### KD with lentiviral shRNAs

For transient KD with lentiviral shRNA, rat hippocampal neurons were transduced with lentiviral particles (shA, Sigma, MISSION Lentiviral Transduction Particle; shB, Dharmacon, SMARTvector Lentiviral shRNA, Table 2) targeting each gene at MOI of 2 (Sigma) or 10 (Dharmacon) on day *in vitro* 7 (DIV7). Non-mammalian gene targeting shRNAs were used as control for each version of lentiviral shRNA. Exact targeting sequence of each lentiviral shRNAs is listed in Table 2. For A555-PFF uptake study, one shRNAs (highlighted in blue in Table 2) were selected for each target KD.

**Table 2:** Sequence of lentiviral shRNA

Gene	Label	Manufacturer	Catalog no.	Clone ID	Target sequence
TMEM175	shA_TMEM175 TMEM175	Sigma	CSTVRS		CCAGCATCTTCCAGTTTGC
	shB_TMEM175-1	GE Healthcare	V3SR7594	V3SVRNHC_23419598	TAGTACAAGTTTATCGCGA
	shB_TMEM175-2	GE Healthcare	V3SR7594	V3SVRNHC_23588129	CATGACATTTCTAATCGTA
	shB_TMEM175-3	GE Healthcare	V3SR7594	V3SVRNHC_24959246	CCAGCAGACCTCGGCGTTT
GAK	shA_GAK-1	Sigma	SHCLNV	TRCN0000321679	TGGCCTGACTGAGGCACAAAT
	shA_GAK-2	Sigma	SHCLNV	TRCN0000321738	CCAGAAATTGTAGACCTGTAT
	shB_GAK-1	GE Healthcare	V3SR7594	V3SVRNHC_19616843	CATGCTAAAGGTCAATCCA
	shB_GAK-2	GE Healthcare	V3SR7594	V3SVRNHC_20845763	CCAACAAGGTGATTGCTTC
	shB_GAK-3	GE Healthcare	V3SR7594	V3SVRNHC_24476357	AGTTCAAGGCGATGTCCTC
IDUA	shB_IDUA-1	GE Healthcare	V3SR7594	V3SVRNHC_18278231	CAGGGCAGGGATTGATCTA
	shB_IDUA-2	GE Healthcare	V3SR7594	V3SVRNHC_22093427	GTATATGCCCCAATCAACA
	shB_IDUA-3	GE Healthcare	V3SR7594	V3SVRNHC_22261166	GAACCACTGTCCTGATCTA
PCGF3	shA_PCGF3	Sigma	SHCLNV	TRCN0000095498	GACTGAGTGTTTGCACACATT
	shB_PCGF3-1	GE Healthcare	V3SR7594	V3SVRNHC_21231731	CCATCAGAGCCACCCGTTA
	shB_PCGF3-2	GE Healthcare	V3SR7594	V3SVRNHC_23670530	TGCGTGGCCTCAAGCGGAA
	shB_PCGF3-3	GE Healthcare	V3SR7594	V3SVRNHC_24942779	GTATATCGGTCATGACAGA
DGKQ	shA_DGKQ	Sigma	SHCLNV	TRCN0000025388	CAAGAGGTTCTCCCACTGTTT
	shB_DGKQ-1	GE Healthcare	V3SR7594	V3SVRNHC_20543285	GTGTACATTTGGACGTCTA
	shB_DGKQ-2	GE Healthcare	V3SR7594	V3SVRNHC_20950736	AGGATCGACAGCACGATTA
	shB_DGKQ-3	GE Healthcare	V3SR7594	V3SVRNHC_24195329	ACGACAGGTTGAGGATCAG
CPLX1	shA_CPLX1-1	Sigma	SHCLNV	TRCN0000338026	GAGCATCCTGGACACTGTCAT
	shA_CPLX1-2	Sigma	SHCLNV	TRCN0000379852	GACTCGACCCAAGAAGGCTA
	shA_CPLX1-3	Sigma	SHCLNV	TRCN0000380794	GGAGGCAGAACGTGAGGTCA
SLC26A1	shB_SLC26A1-1	GE Healthcare	V3SR7594	V3SVRNHC_02515367	ACGTGGCGTATGTATGGTA
	shB_SLC26A1-2	GE Healthcare	V3SR7594	V3SVRNHC_24650069	GCAGAAATGTTTGCACGTA

### Production of AAV8-hTMEM175 WT and p.M393T and transient overexpression

Human TMEM175 tagged with Myc at c-terminal was excised out from RC201422 (Origene) and RC201422 with point mutation that results amino acid 393 from methionine (ATG) to threonine (ACG) by SalI and PmeI digestion and subcloned into a pAAV *cis* plasmid that contains human Synapsin promoter and WPRE. AAV8-hSyn-TMEM175-WPRE was produced by the helper-free triple-plasmid transfection method as described (32). The AAV *cis* plasmid, an adenoviral helper plasmid and a packaging plasmid that encodes the Rep gene from AAV2 and Cap gene from AAV8 were cotransfected into HEK293 cells. AAV vector was subsequently purified by two rounds of cesium chloride density gradient ultracentrifugation. The titers of AAV vectors were determined via qPCR analysis, and the purity of the AAV preps was determined by silver staining. Primary rat hippocampal neurons were transduced with AAV particles on DIV7 at indicated MOI. TMEM175 overexpression positive cells were identified by the detection of MycTag, which distinguishes ectopically expressed TMEM175 from endogenous TMEM175. Percentage of TMEM175 positive cells was calculated by dividing the number of MycTag-positive cells by the total number of healthy nuclei.

### RNA isolation and quantitative real-time PCR (qRT-PCR)

Total RNAs from cells were isolated and reversed transcribed to cDNA using cell-to-ct kit (Applied Biosystems) following the manufacturer's instruction. Gene expression was detected by Taqman gene expression assay.

### **α**-Synuclein PFF seeding and uptake assay

αSyn PFF was prepared according to previously described method (13) using human α-synuclein (rPeptide, S-1001-2). Sonicated α-synuclein PFF (100 μg/ml) was diluted in media and added to neurons (1–4 μg/ml, 0.1 ml/96 well) 4 days after lentivirus or AAV transduction. Neurons were incubated for 14 days post-PFF addition. Approximately 50% of media was replaced with fresh media once a week. For comparison of WT and αSynKO neurons, cells were treated with 0–8 μg/ml PFF and incubated for 7–21 days. To monitor PFF uptake, Alexa Flour 555 (Invitrogen A30007) labeled αSyn was made in to PFF in the same way as unlabeled αSyn. Four days after lentivirus transduction, 4 μg/ml Alexa 555-PFFs in a complete medium were given to rat hippocampal neurons at 37°C, 5% CO2 for indicated hours, the last 15 min of which was also with Hoechst 33342 (Life Technologies; 1:5000) to label nuclei. After incubation, cells were rinsed two times, supplied with fresh media red background suppressor (Life Technologies), and live-cell imaged using ArrayScan™ XTI Live High Content Platform with 20× objective.

### Immunocytochemistry

After rinsing with DPBS, cells were fixed with 4% paraformaldehyde/4% sucrose at room temperature for 15 min. For analysis of LC3 puncta, cells were fixed with 100% methanol for 15 min at −20°C. Cells were then rinsed 3× with DPBS, permeabilized and blocked with permeabilization/blocking buffer (0.2% BSA, 10% Goat serum, 0.2% Tx-100 in DPBS) for 1 h at room temperature. Then, cells were incubated with primary antibodies (βIIItubulin 1:500, Ab78078; pS129-α-syn 1:500, Ab51253; α-synuclein 1:250, Ab27766; MycTag 1:500 Cell signaling 2276; LC3 1:200, Cell signaling 4108S) diluted in blocking buffer (5% Goat serum, 0.2% BSA in DPBS) at 4°C overnight. After rising 2× with 0.05% Tween 20 in DPBS and 1× with DPBS, each well was incubated with matching goat antimouse/rabbit Alexafluor conjugated secondary antibody (1:1000, Life Technologies) and Hoechst 33342 (1:10 000, Invitrogen) in blocking buffer at 1 h at room temperature (RT). After rinsing, antibody-specific fluorescence was visualized with imaging using ArrayScan™ XTI Live High Content Platform (Thermo Fisher) with a 20× objective.

### High-content image analysis

ArrayScan™ XTI Live High Content Platform was used for the high-content imaging. At least 9 fields per well were imaged, analyzed, and averaged. A total of 3–6 wells containing ~20 000 cells per well were used per treatment condition (n = 3–6). Image analyses and calculations were performed using HCS Studio™ Cell Analysis Software (ThermoFisher). Hoechst staining was used to label cell nuclei. For analysis of p-α-syn, neuronal profiling BioApplication where single cell was identified based on total βIII-tubulin staining that marks both the cell cytoplasm and neurites was used. Puncta were determined positive if they presented fluorescence intensity that was higher than a fixed threshold within a given pixel. Total intensity of p-α-syn positive puncta per cell was quantified within defined neurite region stemming out of cell body. For analysis of other puncta including Oregon-Green 488, LC3, and Alexa555-PFF, compartmental analysis BioApplication was used. In this analysis, nucleus detected based on Hoechst staining was enlarged to cover the size of cytoplasm and defined as a region to look for marker-specific puncta. Average intensity per cell within defined region was quantified for Oregon green488 positive puncta. Total intensity per cell was quantified within defined cell region for LC3 positive puncta. Total area per cell was quantified within defined cell region for Alexa555-PFF positive puncta. Each analyzed feature was graphed relative to the value of shCTL or WT within the same plate.

### Cell viability

Cell viability was assessed by counting the number of healthy nuclei stained with Hoechst 33342. Healthy nuclei were selected by filtering out morphologically condensed and compact Hoechst 33342 staining, which indicates apoptotic nuclei.

### Isolation of lysosomal fraction

Cells were washed in PBS, trypsinized, quenched with media, and centrifuged 500*g* for 5 min to produce cell pellets. Pellets were resuspended in sucrose homogenization buffer (0.25 m sucrose, 20 mm HEPES) containing protease inhibitor and phosphatase inhibitor (Cell Signaling) and homogenized by sonication (10 pulses of 0.5 s). Homogenates were centrifuged at 500*g* for 5 min to discard unbroken cell debris. Resulting supernatant (S1) was centrifuged at 6800*g*, 10 min to yield P2. Remaining supernatant (S2) is centrifuged again at 20 000*g* for 30 min to yield P3, which is washed with fresh Sucrose Homogenization buffer and centrifuged at 20 000*g* for 15 min. P2/P3 fractions were resuspended in RIPA buffer containing protease inhibitor and phosphatase inhibitor.

### Lysosomal GBA activity assay

GBA activity was measured using 4-methylumbelliferyl β-d-glucopyranoside (4MU, Sigma). Five micrograms of lysosomal fraction was adjusted to 50 μl per well with activity assay buffer for experimental samples. Fifty microliters of blank activity assay buffer was used for measuring background, and 5 μg lysosomal fraction with 1 mm conduritol-β-epoxide (CBE, Sigma) was used as negative control. Fifty microliters of 4-MU reaction mix with 2 mm 4-MU and 2% BSA in activity assay buffer (Sigma, 0.25% Triton X-100, 0.25% Taurocholic acid, 1 mm EDTA in citrate phosphate buffer [Sigma, 400 mm citric acid, 800 mm Dibasic Na phosphate, pH 5.4]) was added to each well and incubated at 37°C for indicated amounts of time, and fluorescence from converted substrate was measured at Ex/Em = 355/460 nm by Tecan Infinite M1000 plate reader. The first-order rate constant (*k*) of enzymatic reaction was obtained by GraphPad Prism version 7.0 (GraphPad Software) one-phase association fit with shared plateau.

### Immunoblotting analyses

Ten to 20 μg of proteins from lysosomal fraction were separated by 4–12% NuPAGE Bis-Tris gel and transferred to nitrocellulose membrane using iBlot (Invitrogen, P3 7min). Blots were blocked with Odyssey Block Buffer (Licor) for 1 h and probed overnight with primary antibodies (TMEM175 1:500, Proteintech Group 19 925-1-AP; MycTag [AAV TMEM175] 1:1000, Cell signaling 2276; LAMP1 1:500 Abcam, ab24170; GAPDH 1:1000, Abcam, ab9485). Appropriate IRDye-conjugated secondary antibodies (1:10000, Licor) were used to visualize proteins with Odyssey imaging system (Licor).

### Statistical analyses

Statistical analysis was done by the GraphPad Prism version 7.05 (GraphPad Software). Two-way analysis of variance (ANOVA) followed by Fisher's Least Significant Difference (LSD) multiple comparison test was used in the studies that analyze more than two parameters assuming each comparison is independent from others. One-way ANOVA was used for analysis of data from three or more groups. Statistical significance was determined by a *P*-value of less than ^*^0.05, ^**^0.01 and ^***^0.001.

## Supplementary Material

HMG-2019-TWB-00047_R2_Supp_Data_ddz136
