# Supplementary material for: Functionalization of the TMEM175 p.M393T variant as a risk factor for Parkinson disease
Source: Hum Mol Genet. 2019 Jun 7;28(19):3244–54. doi: 10.1093/hmg/ddz136 (PMC6859430; doi:10.1093/hmg/ddz136)
Supplement: HMG-2019-TWB-00047_R2_Supp_Data_ddz136 [file hmg-2019-twb-00047_r2_supp_data_ddz136.docx]

**
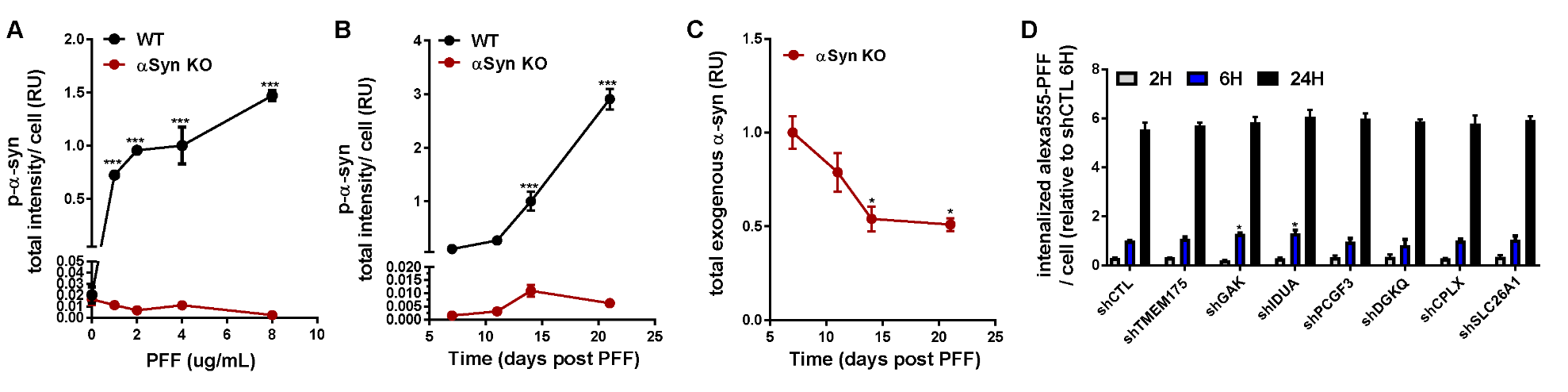
**

**Figure S1. *TMEM175* deficiency alone exacerbates PFF-seeded generation of endogenous phosphorylated α-synuclein inclusions in rat primary hippocampal neurons without changes in PFF uptake.** (A-B) Intensity of p-α-syn inclusion from PFF treated aSyn knock out (KO) and wildtype (WT) as a function of increasing concentration (A) of PFF or time (B) was plotted (n=4). (C) Total amount of exogenous α-syn PFF remaining in aSyn knock out (KO) neurons at each time point was plotted (n=4). (D) The amount of internalized PFF 4 day after shRNA infection against each gene was plotted (n=6-9). Data presented are mean+SEM. Two way ANOVA in A (0ug/mL vs 1, 2, 4, and 8ug/mL in WT and KO) and B (7 days vs 11, 14 and 21 days in WT and KO). One way ANOVA in C (7 days vs. 11, 14 and 21 days) and D (shCTL vs shTargets for 2, 6, and 24H) ** P<0.05, ** P < 0.01 and *** P < 0.001

**
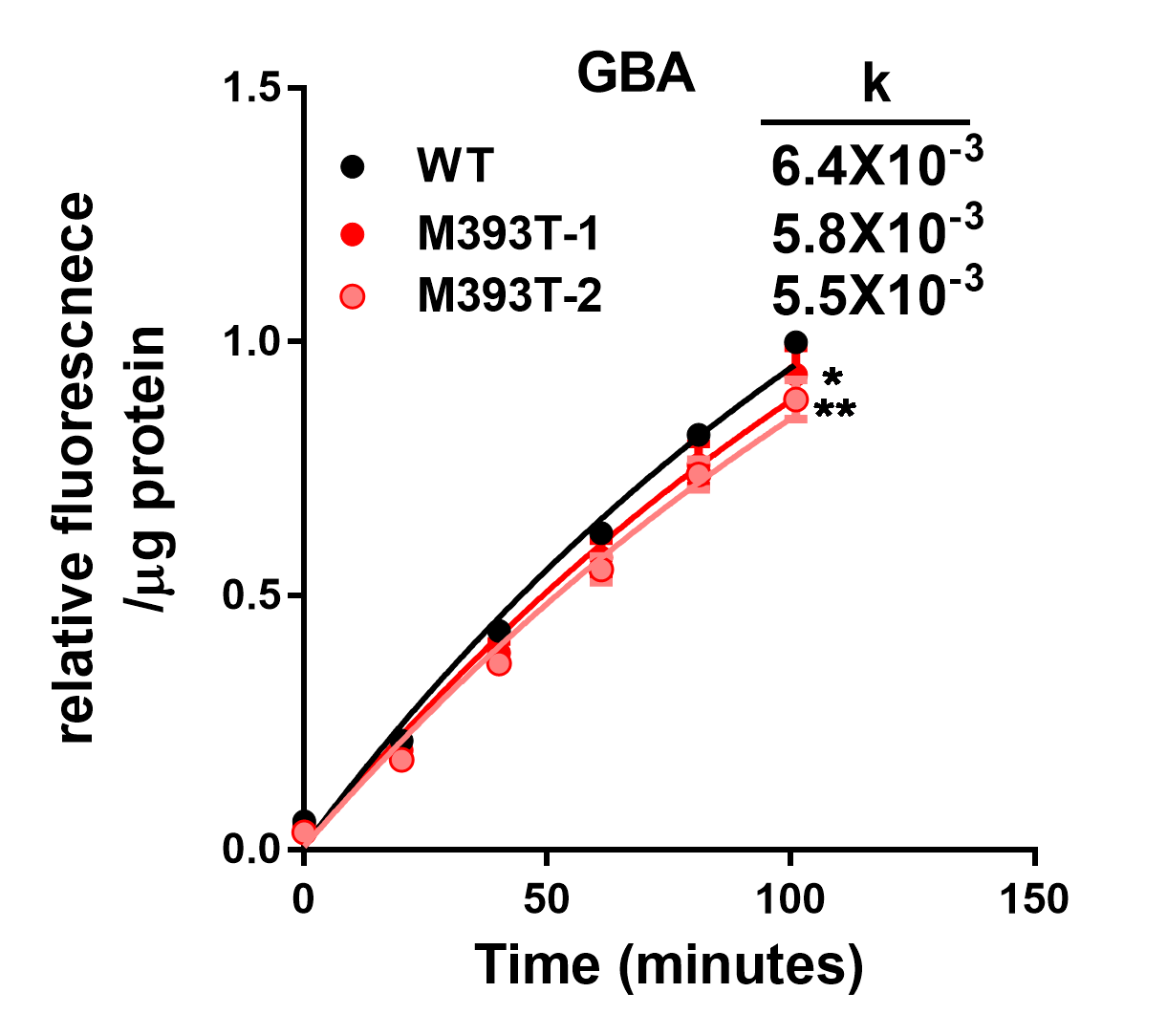
Figure S2. Lysosomal GBA activity is mildly decreased by TMEM175 M393T.** Lysosomal enzyme activity of GBA was determined by the relative fluorescence from enzyme activities of crude lysosomal fractions of WT and TMEM175 M393T cells (*n*=3). Relative fluorescence as a function of time was fit to first order reaction with rate constants, k, for each group indicated on the right. Data are given in relative units (RU). Data presented are mean+SEM. Two way ANOVA **P*<0.05 and ** *P* < 0.01
